# Multidimensional 1-Year Outcomes After Intensive Care Admission for Multisystem Inflammatory Syndrome in Children

**DOI:** 10.1097/CCE.0000000000001213

**Published:** 2025-01-22

**Authors:** Thomas C. Seijbel, Levi Hoste, Corinne M. P. Buysse, Karolijn Dulfer, Filomeen Haerynck, Matthijs de Hoog, Naomi Ketharanathan

**Affiliations:** 1 Department of Neonatal and Pediatric Intensive Care, Division of Pediatric Intensive Care, Erasmus MC Sophia Children’s Hospital, Rotterdam, The Netherlands.; 2 Department of Pediatric Pulmonology, Infectious Diseases and Immune Deficiency, Centre for Primary Immune Deficiency Ghent, Jeffrey Modell Diagnosis and Research Centre, Ghent University Hospital, Ghent, Belgium.

**Keywords:** child, COVID-19, follow-up, multisystem inflammatory syndrome in children, pediatric intensive care unit

## Abstract

**OBJECTIVES:**

The COVID-19 pandemic gave rise to uncertainty concerning potential sequelae related to a severe acute respiratory syndrome coronavirus 2 infection. This landscape is currently unfolding with studies reporting sequelae on various domains (physical, cognitive, and psychosocial), although most studies focus on adults or only one domain. We sought to investigate concurrent sequelae on multiple domains 1 year after PICU admission for Multisystem Inflammatory Syndrome in Children (MIS-C).

**DESIGN:**

Prospective cohort study.

**SETTING:**

Two academic, tertiary referral hospitals in The Netherlands and Belgium.

**PATIENTS:**

Patients (< 18 yr, *n* = 58) seen in-person 1-year after PICU admission for MIS-C.

**INTERVENTIONS:**

None.

**MEASUREMENTS AND MAIN RESULTS:**

Seventy MIS-C patients (62% male; median age, 10.0 [interquartile range, 7.4–13.0]) were admitted to the PICU, mostly (86%) due to (imminent) circulatory failure. The majority received IV immunoglobulins (95%), steroids (83%), and vasopressors and/or inotropes (72%). Invasive respiratory support and extracorporeal membrane oxygenation were necessary in 7% and 2%, respectively. All patients survived. Fifty-eight patients (83%) attended 1-year follow-up. Although most patients had normal functional performance scores (Pediatric Cerebral Performance Category, Pediatric Overall Performance Category, and Functional Status Score), 62% still experienced physical sequelae: fatigue (40%), headaches (27%), and decreased exercise tolerance (19%). Cognitive, behavioral, and psychological problems were reported in 14%, 13%, and 23%, respectively. This resulted in 22% requiring ongoing healthcare utilization, 9% not being able to return to full-time school attendance and cessation of hobbies in 7%.

**CONCLUSIONS:**

This is the first 1-year outcome study of MIS-C PICU patients to include both physical and psychosocial characteristics. One year after PICU admission, most children had normalized functional performance as measured by three validated performance scores. However, many still reported a variety of multidimensional sequelae at 1-year follow-up impacting daily life. This emphasizes the importance of continued investigative efforts and multidisciplinary follow-up programs to better understand pathophysiology and contributing factors to the MIS-C disease trajectory and initiate patient-specific interventions to improve outcome and social participation.

**KEY POINTS**

**Question:** What is the multidimensional outcome (physical, psychosocial) of Multisystem Inflammatory Syndrome in Children (MIS-C) 1 year after intensive care?

**Findings:** A prospective cohort study of 58 children showed mostly normal validated functional scores 1 year afterward. In contrast, 62% reported physical complaints as well as cognitive (14%), behavioral (13%), and psychological (23%) sequelae resulting in ongoing healthcare utilization (22%), decreased school attendance (9%), and hobby cessation (7%).

**Meaning:** MIS-C intensive care patients report multidimensional sequelae 1-year after admission impacting daily life, emphasizing the importance of longitudinal research and multidisciplinary follow-up for improved disease trajectory understanding and patient-specific treatments.

During the COVID-19 pandemic, up to three-quarters of children with Multisystem Inflammatory Syndrome in Children (MIS-C) were admitted to a PICU due to life-threatening multiple organ inflammation (1). Despite potential longstanding effects of this novel disease, the aftermath of MIS-C is understudied, focusing mainly on the short-term and on cardiac or neurological outcomes (2–5). It is known that significant, persistent morbidities in several developmental domains (e.g., neurocognitive and psychological) might emerge in PICU survivors. These morbidities can further impact child development in terms of functional milestones, social interactions, and school development resulting in a cascade of sequelae (6). Furthermore, reports of long-term sequelae of COVID-19 in the adult population raise concerns about the possibility of a similar trajectory occurring in children following a severe acute respiratory syndrome coronavirus 2 infection (7).

We sought to investigate long-term sequelae of MIS-C in multiple domains given the severe systemic inflammatory nature of the disease requiring PICU admission.

## METHODS

Patients with MIS-C (World Health Organization definition [8]), admitted to the PICU prior to May 2022, were seen in-person at 1-year follow-up at Erasmus MC Sophia Children’s Hospital, Rotterdam, The Netherlands or Princess Elisabeth Children’s Hospital, Ghent University Hospital, Belgium. Follow-up was a standard part of care after admission to the PICU for MIS-C due to the novelty of MIS-C. Consent for this study was granted by the Medical Research Ethics Committee Erasmus MC on August 6, 2021, under study number MEC-2021-0538, titled “Outcome after PICU admission for Multisystem Inflammatory Syndrome in Children (MIS-C).” The Medical Research Ethics Committee Ghent University Hospital approved this study on April 6, 2020, under code BC-07574, titled “idiopathische ernstige Covid-19: onderzoek naar de genetische en immunologische basis van ernstige virale infecties”. Procedures were followed in accordance with national laws and the Helsinki declaration. Baseline data were collected prospectively during PICU admission and outpatient visit. **Supplemental Table 1** (http://links.lww.com/CCX/B460) shows the interview list of physical and psychosocial topics and questions asked during outpatient visit. Descriptive statistical analysis of this cohort was performed and reported as percentages for categorical data or median and interquartile range (IQR; first–third quartiles) for nonparametric data. Independent *t* tests were used for assessing differences between patients with and without follow-up data.

## RESULTS

Seventy MIS-C patients were admitted to the PICU. All patients survived. Three- to 6-month outcomes of 21 patients from one of the study sites were included in a previous publication (4). Fifty-eight (83%) were evaluated at 1-year outpatient follow-up. **Supplemental Table 2** (http://links.lww.com/CCX/B460) displays admission parameters of this cohort and available clinical characteristics of patients without consent or follow-up data.

Median follow-up was after 12.9 months (IQR, 11.9–14.1 mo). At follow-up, one patient had persisting arterial hypertension. The sole coronary aneurysm at admission (*z* score = 11.2) had decreased in 9 months (*z* score = 2.8). Echocardiography normalized in all other patients within four months. The rate of overweight (body mass index [BMI] > 1 sd) had slightly increased from 38% (22/58) at PICU admission to 44% (24/55) at follow-up, and the rate of obesity (BMI > 2 sd) from 16% (9/58) to 18% (10/55).

**Figure 1.**
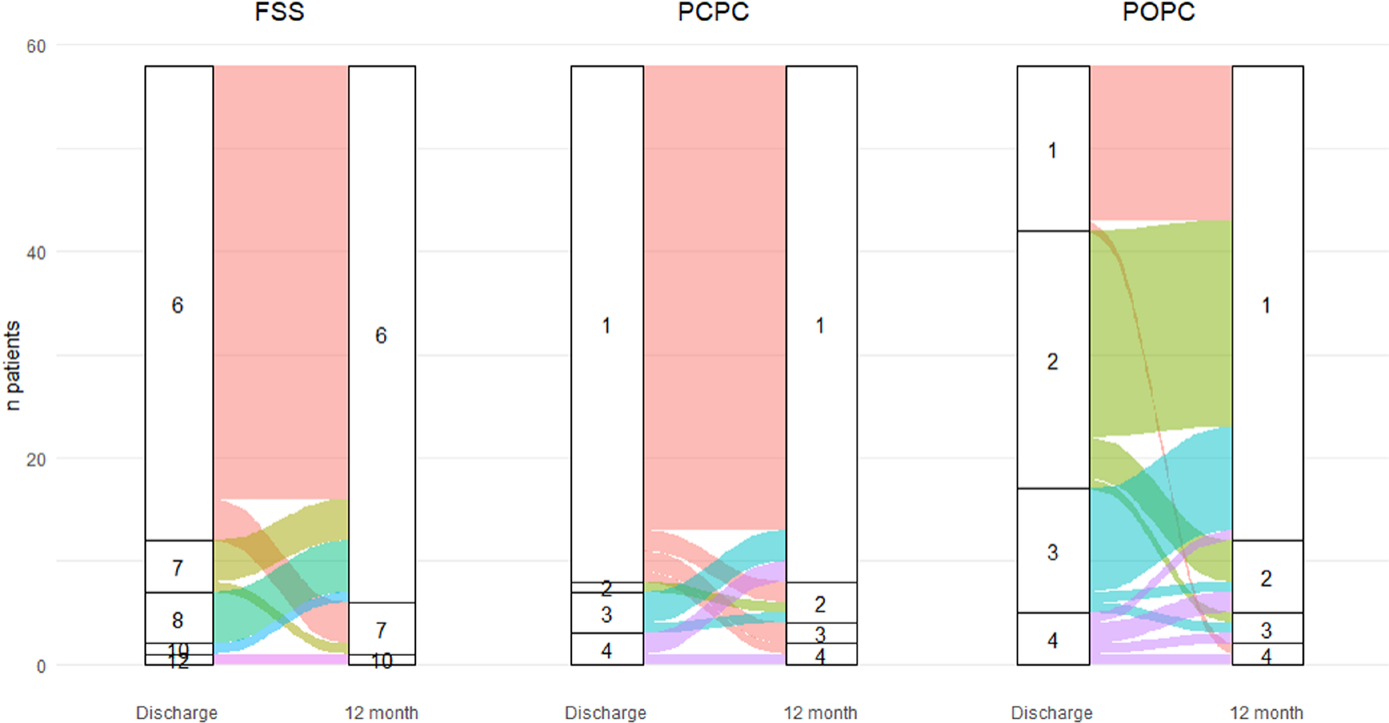
Functional and performance scores at PICU discharge and 1-yr follow-up. Functional Status Score (FSS) ranges from 6 (normal function) to 36 (very severe dysfunction). No patient scored greater than 12. Pediatric Cerebral Performance Category (PCPC) and Pediatric Overall Performance Category (POPC) range from 1 (normal) to 6 (brain death). No patient scored greater than 4.

**Figure 1** displays Functional Status Score, Pediatric Cerebral Performance Category, and Pediatric Overall Performance Category at PICU discharge and at follow-up. These three validated scores were normal at follow-up in most patients.

Although most patients had normal functional performance scores at follow-up, 62% of patients (36/58) reported at least one physical or neuropsychological sequelae since MIS-C. Forty percent (23/58) experienced increased fatigue, 19% (11/58) reported decreased exercise tolerance, and 9% (5/58) cardiopulmonary sequelae (e.g., palpitations, chest pain, shortness of breath). Twenty-seven percent (14/51) reported frequent headaches, impairing daily functioning in two. Twelve children (12/54, 22%) reported an episode of excessive hair loss after hospital discharge within the 1-year follow-up period, which persisted (i.e., decreased hair density and/or more hair loss than before PICU admission) in five children (5/54, 9%). Changes in smell or taste and appetite were present in 2% (1/56) and 5% (3/55). Additionally, cognitive impairments, behavioral problems, and psychological sequelae were reported in 14% (8/57), 13% (7/56), and 23% (13/57), respectively.

As a result, 9% (5/58) had not restarted full-time school attendance and 7% (4/58) had ceased or decreased hobbies. Thirteen patients (22%) remained under care from allied healthcare professionals (dieticians, physiotherapists, psychologists, and social workers) for ongoing sequelae.

## DISCUSSION

This report describes both physical and psychosocial characteristics of MIS-C patients 1 year after PICU admission. Most children had normalized functional performance as measured by three validated performance scores. However, many still reported a variety of multidimensional sequelae at 1-year follow-up impacting daily life. The strengths of this prospective cohort study were the eligibility of all PICU patients with MIS-C admitted in one of two European centers and the high follow-up rate (83%). The high follow-up rate was due to the follow-up program being a standard part of post-PICU outpatient care. The findings in this cohort are therefore likely applicable to other post-PICU MIS-C populations, and shed more light on long-term daily problems experienced by MIS-C intensive care survivors.

Three validated scores were used to objectify functional performance. Despite most children having normal scores, many children (63%) reported a variety of debilitating sequelae. High rates of fatigue, exercise intolerance, and neuropsychological sequelae were present. Nine percent of children had not resumed full-time school attendance and 22% required ongoing healthcare utilization. This underlines how crude functional performance scores insufficiently probe certain clinical and psychosocial sequelae.

Short- to medium-term MIS-C outcome studies (≤ 1 yr) report the heterogeneity of potential sequelae given multiple organ involvement. Examples include decreased exercise tolerance (with or without substantiated standardized physical testing and/or abnormalities on cardiac ultrasound), neurological or cognitive deficits (e.g., fatigue, headaches, muscle weakness, coordination abnormalities, and worse working memory scores), and psychosocial problems with decreased quality of life (2, 4, 5, 9–11). Our findings confirm the diverse nature of MIS-C sequelae. Concerning is the fact self-reported sequelae seem to persist over time and lead to decreased school and social participation.

Major study limitations are potential recall bias and the lack of standardized measures that are validated and/or recommended as part of the PICU Core Outcome Measurement Set (e.g., pediatric quality of life inventory, Patient-reported outcome measurement information system measures) and standardized exertional testing. Incorporation of validated exercise testing in future outcome studies would improve quantification of self-reported physical sequelae. Similarly, the variety of self-reported neuropsychological sequelae reported warrants standardized neurocognitive and psychological testing, as performed at short-to-medium term (3–6 mo) by Otten et al (4). This was not done in this 12-month outcome study because a subgroup of this cohort had already undergone such testing in the short term, and a minimum of the 12-month interval is required (4). Furthermore, the addition of a case-matched control group might help distinguish which reported sequelae are secondary to MIS-C pathophysiology, the critical illness, the PICU experience itself, and COVID-related social measures. The latter is known to have negatively impacted mental and social health in children and adolescents (12).

The multidimensional nature of reported symptoms emphasizes the importance of investigative efforts and longitudinal, multidisciplinary follow-up programs to better understand disease trajectory and contributing factors. This enables early recognition of potentially debilitating sequelae and develop patient-specific interventions. Such an approach is especially important in children, in which the impact of these sequelae in formative years crucial to development could pose lifelong ramifications, not only in future pandemics but also in novel diseases and after pediatric critical illness in general. We therefore advocate increased awareness and ongoing research and healthcare attention for health problems and societal consequences of MIS-C. Lessons learned, of which our findings add to the growing body of knowledge regarding MIS-C, are invaluable for developing appropriate aftercare and counseling.

## Supplementary Material


