# Supplementary material for: Multidimensional 1-Year Outcomes After Intensive Care Admission for Multisystem Inflammatory Syndrome in Children
Source: Crit Care Explor. 2025 Jan 22;7(1):e1213. doi: 10.1097/CCE.0000000000001213 (PMC11756874; doi:10.1097/CCE.0000000000001213)
Supplement: Supplementary file 1 [file cc9-7-e1213-s001.pdf]

Supplemental material

Multidimensional One-year Outcomes after

Intensive Care Admission for MIS-C

Thomas C Seijbel BSc[a], Levi Hoste MD[b], Corinne MP Buysse MD PhD[a], Karolijn Dulfer PhD[a], Filomeen Haerynck MD PhD[b], Matthijs de Hoog MD PhD[a], Naomi Ketharanathan MD PhD[a]

Affiliations: [a] Erasmus MC Sophia Children's Hospital, Department of Neonatal and Pediatric Intensive Care, Division of Pediatric Intensive Care, Rotterdam, The Netherlands; and [b] Department of Pediatric Pulmonology, Infectious Diseases and Immune Deficiency, Centre for Primary Immune Deficiency Ghent, Jeffrey Modell Diagnosis and Research Centre, Ghent University Hospital, Ghent, Belgium.

Address correspondence to: Naomi Ketharanathan MD PhD**,** Department of Neonatal and Pediatric Intensive Care, Division of Pediatric Intensive Care Erasmus MC Sophia Children's Hospital, Wytemaweg 80, Room SK-3232, 3015 CN, Rotterdam, The Netherlands. n.ketharanathan@erasmusmc.nl, [00-31-(0)10-7036189].

Conflict of Interest Disclosures: The authors have no conflicts of interest to disclose.

Funding/Support: No funding was secured for this study.

Key words: Child; COVID-19; follow-up; multisystem inflammatory syndrome in children; pediatric intensive care unit.

Table of contents **Page**

1. Supplemental Table 1 2
2. Supplemental Table 2 3

Supplemental Table 1. One-year post-PICU admission interview

Outcome category	Question	Comparison
**Physical complaints**		
	Fatigability<br>• *Do you need to rest or sleep during daytime?*<br>• *Do you need to rest or sleep after performing a (physical)task?*<br>• *Do you sleep longer in general?*	Individual pre-PICU
	Exercise intolerance<br>• *Are you able to perform the same physical tasks (e.g. bicycle to school, play outside, perform sports)?*<br>• *Can you still participate in physical education at the same level?*	Individual pre-PICU
	Complaints during exercise<br>• *Chest pains?*<br>• *Palpitations?*<br>• *Syncope?*<br>• *Shortness of breath?*	Individual pre-PICU
	Headaches<br>• *Do you experience headaches since admission?*<br>• *Is their frequency more than before MIS-C?*<br>• *Is the aspect different than before MIS-C?*<br>• *Do you experience impairments due to headaches (e.g. not able to attend school)?*	Individual pre-PICU
	Other<br>• *Did you experience hair loss in the past year?*<br>• *Do you still experience increased hair loss of altered hair density?*<br>• *Do you have an altered sense of smell or taste?*<br>• *Do you have an altered appetite?*	Individual pre-PICU
**Neuropsychological complaints**		
	Changes in behavior<br>• *Are there conflicts (family, friends, school)?*<br>• *Is there disruptive and/or aggressive behavior (verbal/physical)?*<br>• *Are you more withdrawn (at home, at school)?*	Individual pre-PICU
	Changes in psychological well-being<br>• *Do you feel more sad or worried?*<br>• *Do you have more nightmares and/or sleep interruptions?*	Individual pre-PICU
	Changes in cognitive functioning<br>• *Do you have problems concentrating (e.g. reading a book, watching television, performing school tasks?)*<br>• *Do you need more time to complete school tasks?*<br>• *Has school level changed after admission?*<br>• *Did you have to repeat your grade?*	Individual pre-PICU
**Social functioning**		
	School attendance	Individual pre-PICU
	Change of hobbies	Individual pre-PICU
	Care from allied health professionals	Individual pre-PICU

The one-year post-PICU admission interview with patient and at least one parent/caregiver was conducted with a pediatric intensivist and a pediatric psychologist as part of the neuropsychological intake. All questions were asked in the presence of both patient and parent/caregiver. Depending on the child's age (>8 years old) or the child's developmental stage, questions were child and/or parent-reported.

Supplemental table 2. Demographic and clinical characteristics at PICU admission

**Variable**	**Inclusions, N[a]**	**N *(%)* or Median *[IQR]***	**Patients not in follow-up and/or no consent, N[b]**	**N *(%)* or Median *[IQR]***
PICU admissions		58 *(100)*		12 *(100)*
Sex, Male		36 *(62)*		7 *(58)*
Age, years		10.0 *[7.4 – 13.0]*		7.5 *[6.3 – 13.1]*
Overweight (BMI>1SD)		22 *(38)*	11	5 *(45)*
Obesity (BMI>2SD)		9 *(16)*	11	3 *(27)*
**Symptoms at Admission**				
Shock		31 *(53)*		N.A.
Gastro-intestinal Symptoms[c]		58 *(100)*		N.A.
Mucocutaneous Erythema		42 *(72)*		N.A.
Fever		58 *(100)*		N.A.
Neurological Symptoms[d]		36 *(62)*		N.A.
Other[e]		3 *(5)*		N.A.
**PICU admission indication**				
(Imminent) circulatory failure		50 *(86)*		12 *(100)*
Respiratory failure		1 *(2)*		0 *(0)*
Other[f]		7 *(12)*		0 *(0)*
**Peak Laboratory Values (Reference Range)**				
CRP (<10 mg/L)	56	198 *[148 – 301]*		263 *[176 – 310]*
ESR (<15 mm/h)	26	68 *[32 – 106]*		N.A.
Ferritin (30-240 µg/L)	57	807 *[515 – 1505]*	12	662 *[459 – 1204]*
IL-6 (<10 pg/mL)	44	145 *[55 – 541]*		N.A.
Procalcitonin (<0.046 µg/L)	27	19.84 *[4.08 – 29.60]*		N.A.
TNF-alpha (<15 pg/mL)	10	31.30 *[19.40 – 129.37]*		N.A.
D-dimers (<500 g/mL)	48	3515 *[2108 – 5468]*		N.A.
PT-INR (0.8-1.2)	53	1.27 *[1.11 – 1.41]*		N.A.
NT-pro-BNP (<127 ng/L)	41	9184 *[4495 – 19033]*	7	5333 [3138 – 34972]
Troponin-T (<0.014 µg/L)	47	0.077 *[0.027 – 0.198]*	10	0.071 [0.022 – 0.197]
**Echocardiography**				
Fractional Shortening (%)	53	27.0 *[24.0 – 31.5]*	11	27.0 *[25.5-35.0]*
Fractional Shortening (Z-score)	47	-3.0 *[-4.0 – -1.2]*		N.A.
**Treatment**				
Intravenous Immunoglobulins		55 *(95)*		N.A.
Steroids		48 *(83)*		N.A.
Adrenalin		7 *(12)*		N.A.
Dobutamine		7 *(12)*		N.A.
Dopamine		1 *(2)*		N.A.
Ephedrine		1 *(2)*		N.A.
Milrinone		34 *(59)*		N.A.
Noradrenalin		25 *(43)*		N.A.
Any Vasopressor or Inotropic		42 *(72)*		N.A.
Peak Vasoactive-Inotropic Score		5.0 *[0.0-13.5]*	11	10.0 *[10.0 – 13.0]*
Invasive Respiratory Support		4 *(7)*		
ECMO		1 *(2)*		
**PICU stay (days)**		3 *[2 – 4]*	11	4 *[3 – 7]*
**Post-PICU hospital stay (days)**		4 *[3 – 6]*	11	4 *[1-5]*

Abbreviations: CRP = C-reactive protein, ECMO = extracorporeal membrane oxygenation, ESR = erythrocyte sedimentation rate, IL-6 = interleukin 6, NT-pro-BNP = N-terminal pro-B-type Natriuretic peptide, PICU = pediatric intensive care unit, PT-INR = prothrombin time, international normalized ratio, TNF-alpha = tumor necrosis factor alpha.

[a] *Number of tested patients; only shown if it is not equal to the total amount of patients in follow-up (N_{total}=58);*

[b] *Number of tested patients; only shown if it is not equal to the total amount of patients not in follow-up or consent (N_{total}=12);*

[c] *Abdominal pain, diarrhea, nausea and/or vomiting;*

[d] *Confusion, convulsions, encephalopathy, Glasgow Coma Scale value <15, headaches and/or neurological deficits;*

[e] *Cervical adenitis (n=1), epididymitis (n=1) and painful scrotal hyperemia (n=1).*

[f] *Acute Kidney Injury (n=1), pain and lethargy (n=1), inability to gain venous access (n=1) and MIS-C, no other indication (n=4).*
